# Tuning visible-light-driven photocatalytic activity of PrMnO_3_ perovskites for tetracycline degradation: mechanistic insights and performance analysis

**DOI:** 10.1039/d6ra06709c

**Published:** 2026-08-12

**Authors:** Lipika Nayak, Tushar Kanta Hati, Akash Panda, Soumyajit Patra, Nigamananda Das, Seemita Banerjee, Purnendu Parhi

**Affiliations:** a Department of Chemistry, Ravenshaw University Cuttack Odisha India pparhi@ravenshawuniversity.ac.in +91-8895193144; b Department of Chemistry, KIIT Deemed to be University Bhubaneswar Odisha India; c Department of Chemistry, Utkal University Bhubaneswar Odisha India; d Chemistry Division, Bhabha Atomic Research Centre Mumbai Maharashtra India

## Abstract

A praseodymium-based perovskite (PrMnO_3_) was synthesised *via* a simple sol–gel route and investigated as a visible-light-active photocatalyst for the degradation of tetracycline. Compared to La, Ce, and Nd based systems, Pr-based materials have received relatively less attention in antibiotic removal, which forms the basis of this study. The structural and physicochemical properties were comprehensively analysed using XRD, FTIR, Raman, FESEM-EDX, XPS, BET, zeta potential, UV-vis DRS, Mott–Schottky, EIS and PL techniques. XRD confirmed the formation of an orthorhombic perovskite phase (*Pnma*) with an average crystallite size of ∼19.8 nm, while spectroscopic analyses verified the presence of MnO_6_ octahedra. The material exhibited a porous and agglomerated morphology with a relatively high surface area (70.375 m^2^ g^−1^). Optical characterisation indicated a band gap of ∼2.23 eV, while Mott–Schottky analysis confirmed the n-type semiconducting nature of the material with a flat-band potential of 0.83 V *vs.* NHE. XPS results revealed mixed-valence states (Pr^3+^/Pr^4+^ and Mn^3+^/Mn^4+^) and oxygen vacancies, which are beneficial for charge separation. The photocatalytic performance of PrMnO_3_ under visible light showed a maximum tetracycline degradation efficiency of 91.04% (*k* = 0.016 min^−1^) within 120 min at near-neutral pH (6.92) in deionised water, with slightly lower efficiency observed in real water matrices (river and tap water). Also, the catalyst maintained excellent stability and reusability, with negligible structural changes after repeated cycles, as evidenced by XRD and FTIR analyses. The reaction followed pseudo-first-order kinetics. Mechanistic insights obtained from LC-MS analysis, radical scavenging experiments, and band structure evaluation reveal that the degradation process is primarily governed by hydroxyl radicals (˙OH) and photogenerated holes (h^+^), while electrons play a crucial role in facilitating charge separation and sustaining the photocatalytic cycle. Overall, the results demonstrate that PrMnO_3_ is a promising and efficient photocatalyst for tetracycline removal under visible-light irradiation.

## Abstract 2

A praseodymium-based perovskite (PrMnO_3_) was synthesised *via* a simple sol–gel route and investigated as a visible-light-active photocatalyst for the degradation of tetracycline.

## Graphical abstract

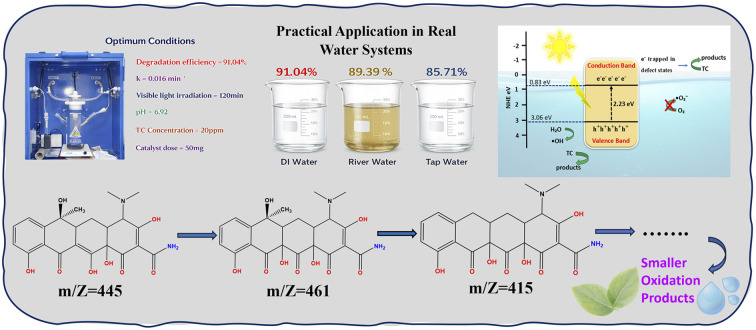

## 1. Introduction

In recent decades, rapid industrial growth, urban expansion, and increasing population pressure have led to a substantial rise in the discharge of organic pollutants such as dyes, phenols, pesticides, surfactants, and pharmaceutical residues into aquatic environments.^1,2^ Among these contaminants, antibiotics have become a serious global concern because they persist in water, can harm ecosystems, and contribute to the development of antibiotic-resistant bacteria. Tetracycline (TC), a widely used broad-spectrum antibiotic in human healthcare and animal farming, is particularly problematic due to its high chemical stability, low biodegradability, and tendency to accumulate in natural water systems. Reports suggest that up to 90% of the administered TC dose is excreted unmetabolized,^3,4^ which explains its frequent detection in rivers, groundwater, and sediments. Its prolonged presence not only promotes the proliferation of resistant bacterial strains but also exerts toxic effects on aquatic organisms and humans, including gastrointestinal distress and renal dysfunction. Therefore, the development of efficient, sustainable, and cost-effective strategies for the degradation of tetracycline and related pharmaceutical pollutants is of urgent importance.^5–8^

Conventional treatment methods such as thermal oxidation, chlorination, Fenton processes, and electrochemical oxidation have shown partial effectiveness but often suffer from high operational costs, complex reaction conditions, and incomplete mineralisation of organic compounds.^9,10^ In this context, photocatalytic degradation driven by visible light has gained immense attention as a sustainable and environmentally benign approach for wastewater purification. In this process, visible-light irradiation promotes electrons from the valence band (VB) to the conduction band (CB) of the semiconductor photocatalyst, resulting in the generation of electron–hole pairs. These charge carriers subsequently participate in surface redox reactions to produce reactive oxygen species (ROS), including hydroxyl (˙OH) and superoxide (˙O_2_^−^) radicals, which play a crucial role in pollutant degradation.^11–13^ The generated ROS can further break down complex antibiotic molecules into harmless end products such as CO_2_ and H_2_O, making this a clean and energy-efficient remediation strategy.

Among various semiconductor materials, rare-earth-based perovskite oxides (REMO_3_, where RE = rare-earth element and M = transition metal) have emerged as promising candidates for visible-light-driven photocatalysis. Their exceptional photocatalytic activity stems from narrow, tunable band gaps, high thermal and chemical stability, and flexible redox behaviour arising from multiple oxidation states. For example, LaFeO_3_/MgFe_2_O_4_ synthesised by the sol–gel route achieved 97.01% degradation of TC (300 mg L^−1^) under 300 W Xe lamp irradiation within 120 min,^14^ while ZnIn_2_S_4_/LaFeO_3_ composites completely degraded 50 mg per L TC under a 100 W halogen lamp within 40 min.^15^ Likewise, Ce- and Sm-based perovskite composites such as TiO_2_/CeO_2_/CeFeO_3_,^16^ ZnO/CeO_2_/CeFeO_3_,^17^ and g-C_3_N_4_/SmFeO_3_ (ref. 18) have demonstrated >95% degradation efficiency of tetracycline under visible-light irradiation. These reports collectively highlight the remarkable potential of rare-earth perovskite systems for antibiotic degradation.

Despite considerable progress in perovskite photocatalysts, praseodymium-based systems, particularly PrMnO_3_, remain relatively unexplored for photocatalytic wastewater remediation.^19^ The presence of Pr^3+^ ions with a 4f^2^ electronic configuration can influence the electronic structure of the perovskite lattice by creating localised states within the band gap, which helps broaden visible-light absorption and improve charge-carrier separation.^20^ In addition, the coexistence of Mn^3+^/Mn^4+^ oxidation states supports reversible redox cycling,^21^ thereby enhancing the generation of reactive oxygen species involved in pollutant degradation. The PrMnO_3_ lattice can also accommodate oxygen vacancies that act as active centres for oxygen adsorption and activation, further contributing to improved photocatalytic performance.^22,23^ Moreover, its orthorhombic crystal structure offers good structural robustness and reusability, which are desirable features for practical environmental applications.

Current research on rare-earth-based perovskite photocatalysts has primarily focused on La-, Ce-, and Nd-based systems. In contrast, the visible-light-driven photocatalytic performance of pristine PrMnO_3_ for antibiotic degradation remains largely unexplored. To address this research gap, the present work reports the synthesis of phase-pure PrMnO_3_ nanorods *via* a facile sol–gel route and systematically correlates their structural, surface, optical, and electronic properties with photocatalytic performance. The synthesised PrMnO_3_ exhibits a high specific surface area (70.375 m^2^ g^−1^), a mesoporous structure with an average pore diameter of 4.694 nm, and a narrow optical band gap of 2.23 eV, which are favourable for visible-light photocatalysis. XPS analysis further reveals the coexistence of mixed-valence Pr^3+^/Pr^4+^ and Mn^3+^/Mn^4+^ species together with abundant oxygen vacancies, facilitating efficient charge separation. Under the optimized reaction conditions, pristine PrMnO_3_ achieved 91.04% tetracycline degradation within 120 min under visible-light irradiation. The photocatalytic performance was systematically evaluated through operational parameter optimisation, kinetic analysis, recyclability assessment, and testing in different water matrices. Furthermore, electrochemical impedance spectroscopy, steady-state photoluminescence, active-species trapping, Mott–Schottky analysis, and LC-MS were employed to elucidate the charge-transfer behaviour and photocatalytic degradation mechanism. This study demonstrates the potential of pristine PrMnO_3_ as an efficient visible-light-responsive photocatalyst for antibiotic remediation while providing new insights into the structure property performance relationship of underexplored Pr-based perovskites.

## 2. Experimental section

### 2.1. Chemicals and materials

All chemicals used in this work were of analytical grade and were used as received without additional purification. Tetracycline (I.P., 500 mg capsule) was purchased from Abbott India Limited. Praseodymium nitrate hexahydrate (Pr(NO_3_)_3_·6H_2_O, ≥99% purity), manganese nitrate tetrahydrate (≥98% purity), citric acid (≥99.5% purity), hydrochloric acid (HCl, 37%, AR grade), and sodium hydroxide (NaOH, ≥98% purity) were obtained from Sisco Research Laboratories (SRL) Pvt. Ltd. Deionised water was used throughout the experiments as the solvent and reaction medium for the photocatalytic degradation studies.

### 2.2. Synthesis of PrMnO_3_

PrMnO_3_ nanoparticles were prepared through a sol–gel route using citric acid as both the chelating agent and fuel source. A 0.1 M aqueous solution of praseodymium nitrate [Pr(NO_3_)_3_·6H_2_O] and a 0.1 M aqueous solution of manganese nitrate [Mn(NO_3_)_2_·4H_2_O] were prepared separately. Subsequently, 15 mL of each precursor solution (corresponding to 1.5 mmol of Pr^3+^ and Mn^2+^ ions, respectively) was mixed and homogenised under continuous magnetic stirring at 80 °C to ensure uniform dispersion of the metal ions. An equimolar amount of citric acid (3 mmol), dissolved in distilled water, was then added dropwise to the mixed solution. Citric acid coordinates with the metal ions through its carboxylate groups, forming stable metal–citrate complexes that prevent phase segregation and promote molecular-level homogeneity. Upon further heating under continuous stirring at 180 °C, polycondensation and polyesterification reactions occurred, resulting in the formation of a viscous polymeric gel.

**Fig. 1:**
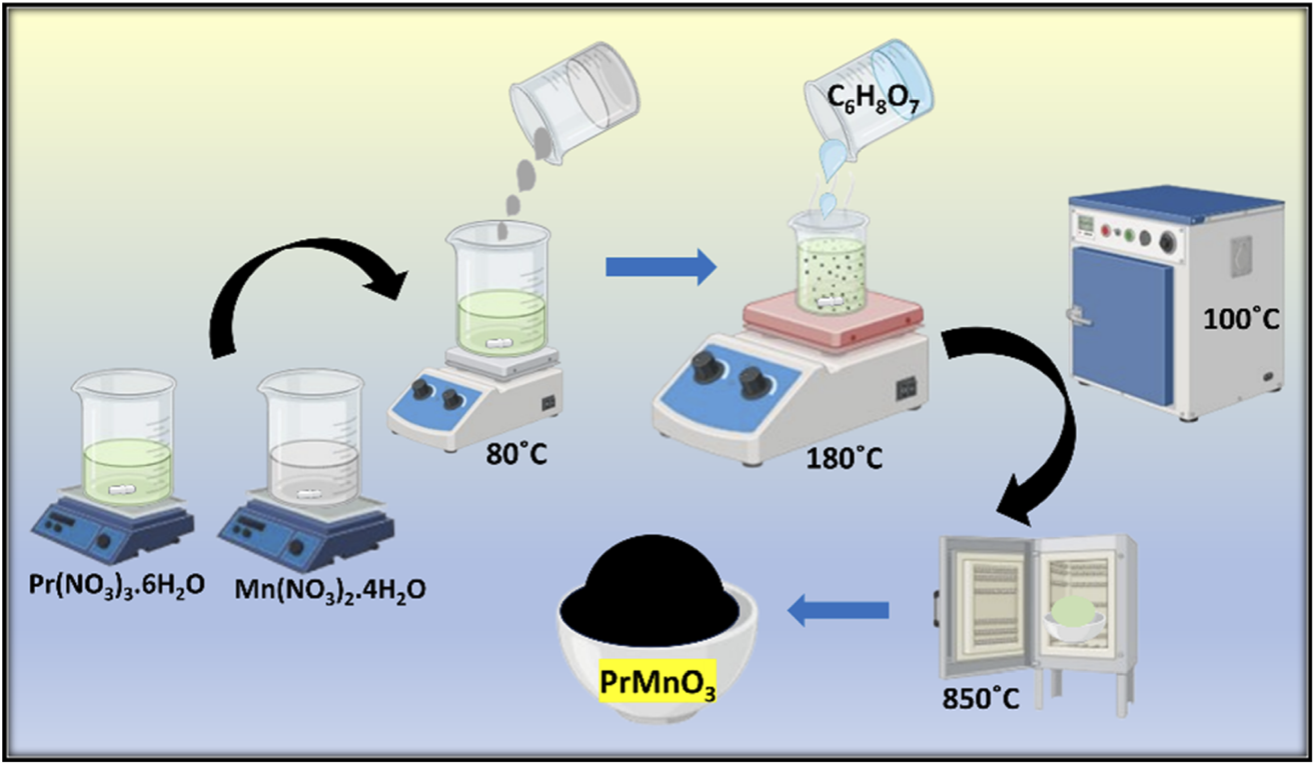
Schematic representation of the sol–gel synthesis procedure for PrMnO_3_ nanoparticles.

The obtained gel was subsequently dried in a hot-air oven at 100 °C for 12 h to eliminate residual solvent and enhance network crosslinking. The dried gel was then calcined at the optimised temperature of 850 °C. The calcination temperature of 850 °C was selected based on prior literature reports that demonstrate the formation of phase-pure orthorhombic PrMnO_3_ under similar synthesis conditions. This temperature provides sufficient thermal energy for complete precursor decomposition and crystallisation while minimising excessive particle growth. During calcination, the metal–citrate complexes decomposed through a self-combustion process, in which nitrate ions acted as oxidising agents while citric acid served as the fuel. This exothermic process facilitated the formation of intermediate metal oxides, followed by crystallisation and solid-state diffusion of Pr–O and Mn–O species, ultimately yielding phase-pure orthorhombic PrMnO_3_ nanoparticles. The evolution of gaseous by-products (CO_2_, H_2_O, and NO_*x*_) during calcination helped restrict particle growth and generate porous nanostructured PrMnO_3_. A schematic illustration of the sol–gel synthesis route for PrMnO_3_ nanoparticles is presented in Fig. 1.

### 2.3. Physical characterisation of PrMnO_3_

The as-prepared PrMnO_3_ catalyst was thoroughly characterised using a range of analytical techniques to investigate its structural, functional, morphological, surface, and optical properties. The crystal structure and phase purity were analysed by X-ray diffraction (XRD) using a Rigaku Advance ULTIMA IV diffractometer with Cu Kα radiation (*λ* = 1.5406 Å), recorded over a 2*θ* range of 10°–80° at a scan rate of 4° min^−1^. Fourier transform infrared (FTIR) spectroscopy was performed on a Thermo Scientific Nicolet spectrometer in the wavenumber range of 400–4000 cm^−1^ using KBr pellets to identify surface functional groups and metal–oxygen bonding vibrations. The surface morphology and microstructural characteristics were examined by field-emission scanning electron microscopy (FESEM, Zeiss Gemini 300). For FESEM analysis, the powdered samples were fixed on carbon tape, coated with a thin layer of gold, and imaged to study particle shape, agglomeration, and size distribution. Textural properties were evaluated from nitrogen adsorption–desorption isotherms measured at 77 K using a BELSORP-mini analyser. The specific surface area was calculated by the Brunauer–Emmett–Teller (BET) method, while pore size distribution and total pore volume were determined using the Barrett–Joyner–Halenda (BJH) model. The surface charge behaviour and colloidal stability of PrMnO_3_ in aqueous suspension were determined by zeta potential measurements using a Nano Zetasizer (HORIBA Scientific, Japan), providing useful information on surface interactions during adsorption and photocatalytic processes. Raman spectra were recorded using a RENISHAW InVia Raman microscope to examine lattice vibrations, crystallinity, and structural distortions associated with Pr–O and Mn–O bonds. The elemental composition and oxidation states of the surface species were investigated by X-ray photoelectron spectroscopy (XPS) using a PHI 5000 Versa Probe III instrument. Before XPS measurements, the samples were ultrasonically washed with ethanol, dried, freeze-dried to preserve structural features, and pelletised using a hydraulic press. The pellets were then mounted on adhesive copper tape and introduced into the chamber under high vacuum conditions. The optical behaviour of PrMnO_3_ was studied using UV-visible diffuse reflectance spectroscopy (UV-DRS) with an integrating sphere, employing BaSO_4_ as the reference material. The optical band gap was estimated from the absorption edge using the relation *E*_g_ = 1240/*λ*, where *λ* represents the wavelength (nm) at the absorption onset. Liquid chromatography-mass spectrometry (LC-MS) measurements were carried out using an Agilent 6530 Q-TOF LCMS system.

### 2.4. Photocatalytic degradation experiments

Photocatalytic degradation experiments were carried out in a custom-fabricated vertical photoreactor (LELESIL, India) with an adjustable working volume of 25–500 mL. Visible-light irradiation was provided by a 450 W lamp with a manufacturer's specified emission range of 420–700 nm. In a typical run, 100 mL of aqueous tetracycline (TC) solution having an initial concentration of 20 ppm was combined with 50 mg of PrMnO_3_ photocatalyst. The suspension was magnetically stirred under dark conditions for 30 min to attain adsorption–desorption equilibrium between TC molecules and the catalyst surface. After this equilibration step, visible-light irradiation was applied to initiate the photocatalytic reaction. At predetermined intervals, 5 mL samples were collected from the reaction mixture and centrifuged for 5 min to remove suspended catalyst particles. The degradation of TC was monitored from the decrease in absorbance intensity at its characteristic wavelength of 357 nm using a Cary-series UV-vis spectrophotometer (Agilent Technologies, USA), and the corresponding degradation efficiency was determined

To evaluate the effect of operating conditions on photocatalytic performance, a series of experiments was carried out by varying the initial TC concentration (10–50 ppm), catalyst dosage (5–60 mg of PMO), and solution pH. The pH values (2–12) were adjusted using HCl and NaOH solutions. In addition, the photocatalytic activity was examined in different water matrices, including tap water and river water, to assess the practical applicability of the catalyst. The durability and reusability of the prepared photocatalyst were further investigated through repeated cycling tests.

The degradation efficiency was calculated using eqn (1):^4^1
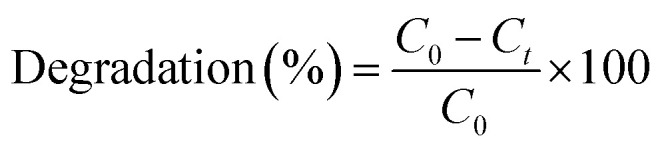
where *C*_0_ and *C*_*t*_ represent the initial TC concentration and the concentration at irradiation time *t*, respectively. To identify degradation intermediates and elucidate reaction pathways, reaction aliquots were centrifuged to remove the photocatalyst, and the resulting supernatants were analysed by liquid chromatography-mass spectrometry (LC-MS).

## 3. Results and discussion

### 3.1. Physicochemical characterisation of PrMnO_3_

#### 3.1.1. Structural analysis (XRD)

**Fig. 2:**
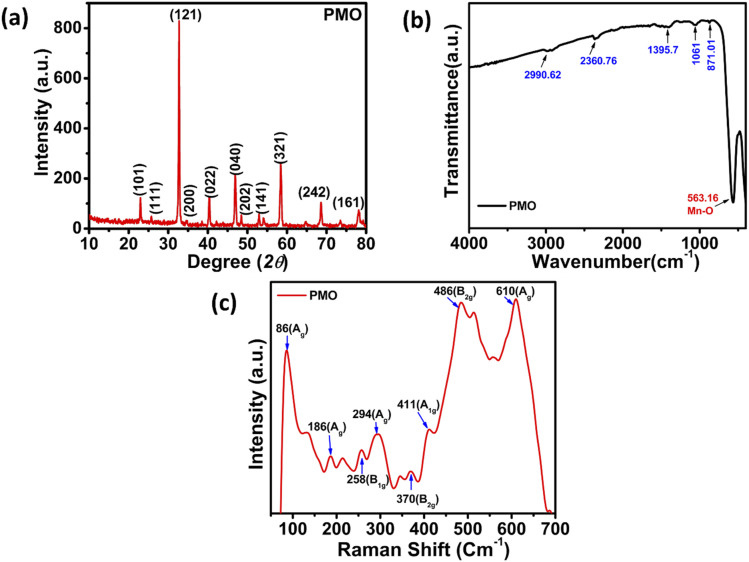
Structural and spectroscopic characterisation of PrMnO_3_: (a) XRD pattern showing orthorhombic phase with indexed planes, (b) FTIR spectrum indicating characteristic Mn–O vibration, and (c) Raman spectrum displaying phonon modes of the *Pnma* structure.

The XRD pattern of PrMnO_3_ displays (Fig. 2a) a series of well-defined diffraction peaks at 2*θ* values of 22.97°, 32.75°, 40.37°, 46.92°, 52.95°, 54.10°, 58.44°, 68.56°, and 78.06°, corresponding to interplanar spacings (*d*) of 3.868, 2.733, 2.233, 1.935, 1.728, 1.694, 1.578, 1.368, and 1.223 Å, respectively. All observed reflections can be indexed to the orthorhombic perovskite structure of PrMnO_3_ (space group *Pnma*) and show good agreement with the standard JCPDS/ICDD data (card no. 85-2203),^22^ confirming the formation of a crystalline single phase without detectable secondary impurities. The most intense diffraction peak at 2*θ* ≈ 32.75°, corresponding to the (121) plane, indicates preferential orientation along this crystallographic direction.

The average crystallite size, calculated from the full width at half maximum (FWHM) of the prominent (121) diffraction peak using the Scherrer equation, was found to be approximately 19.8 nm, confirming the nanocrystalline nature of the material. The interplanar spacing calculated for the prominent reflection using Bragg's law is ∼2.75 Å, which is consistent with reported values for orthorhombic PrMnO_3_ prepared *via* sol–gel and related synthesis routes. The sharp, well-resolved diffraction peaks indicate good crystallinity, which is beneficial for efficient charge transport and enhanced photocatalytic performance.

#### 3.1.2. Functional group analysis (FTIR)

After confirming the crystal structure through XRD analysis, FTIR spectroscopy was carried out to examine the lattice vibrations and surface chemical characteristics of PrMnO_3_ associated with its photocatalytic performance (Fig. 2b). The prominent absorption band observed at ∼563 cm^−1^ is attributed to the Mn–O stretching vibration of MnO_6_ octahedra, confirming the formation of the perovskite ABO_3_ framework, in good agreement with previously reported PrMnO_3_ synthesised *via* sol–gel and combustion routes. The slight broadening of this band reflects the orthorhombic lattice distortion characteristic of rare-earth manganites, which can facilitate charge carrier transport through Mn^3+^/Mn^4+^ redox transitions. The band appearing at ∼1395 cm^−1^, together with the accompanying band at ∼871 cm^−1^, is assigned to the asymmetric stretching and out-of-plane bending modes of surface carbonate (CO_3_^2−^) species, formed due to the adsorption of atmospheric CO_2_ on the highly reactive surface of nanosized PrMnO_3_. The presence of these carbonate species indicates a defect-rich surface, commonly associated with oxygen vacancies in perovskite oxides. Such surface defects play a crucial role in photocatalysis by acting as electron-trapping centres and promoting the activation of molecular oxygen, thereby generating reactive oxygen species (˙O_2_^−^ and ˙OH). The weak absorption band at ∼1061 cm^−1^ is attributed to residual C–O stretching vibrations and/or trace N–O species originating from the nitrate–citric acid sol–gel precursor system. The low-intensity band at ∼2990 cm^−1^ corresponds to C–H stretching vibrations of residual organic fragments, while the feature observed near ∼2360 cm^−1^ arises from atmospheric CO_2_ present in the instrument optical path.^24,25^ The dominance of the Mn–O lattice vibration over residual organic-related bands confirms effective precursor decomposition and high phase purity, consistent with the XRD results. Overall, the well-developed MnO_6_ octahedral network and surface defect states, as evidenced by FTIR, are expected to enhance visible-light absorption, charge-separation efficiency, and interfacial redox reactions, thereby contributing to improved photocatalytic performance of PrMnO_3_.

#### 3.1.3. Raman analysis

In Fig. 2c, the Raman spectrum of PrMnO_3_ exhibits several well-defined phonon modes characteristic of an orthorhombic perovskite lattice belonging to the *Pnma* space group. Group-theoretical analysis predicts 24 Raman-active modes (7A_g_ + 7B_1g_ + 5B_2g_ + 5B_3g_). However, only a limited number of modes are experimentally resolved due to peak overlap, weak intensity, and instrumental limitations. The low-wavenumber band observed at ∼86 cm^−1^ is attributed to A_g_-type external vibrations involving translational motion of Pr^3+^ ions against the MnO_6_ framework. Features appearing in the range 180–300 cm^−1^ originate mainly from octahedral tilting and mixed bending modes, where the peaks near 186 and 294 cm^−1^ are assigned to A_g_ symmetry, while the mode around 258 cm^−1^ corresponds to B_1g_ vibration. In the intermediate region, the band located at ∼370 cm^−1^ is associated with B_2g_ bending of oxygen atoms, whereas the mode near 411 cm^−1^ arises from additional A_g_-type distortions of the octahedral framework. The high-frequency region is dominated by internal Mn–O stretching vibrations of MnO_6_ octahedra. The band observed near 486 cm^−1^ can be assigned to the B_2g_ antisymmetric stretching mode associated with Jahn–Teller distortion of Mn^3+^ ions, whereas the strong peak around 610 cm^−1^ is attributed to the A_g_ symmetric breathing mode of the MnO_6_ octahedra.^26,27^ These Raman characteristics are consistent with earlier reports on rare-earth manganites and further verify the successful formation of phase-pure PrMnO_3_ without any detectable secondary impurity phases.

#### 3.1.4. Morphological and elemental analysis by FESEM-EDX

**Fig. 3:**
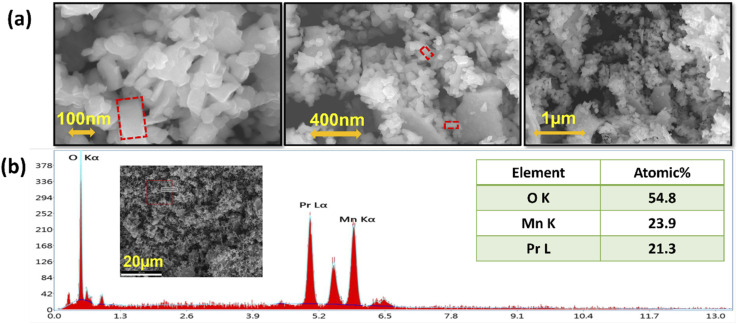
(a) FESEM images of PrMnO_3_ at different magnifications and (b) corresponding EDX spectrum confirming the presence of Pr, Mn, and O.

Field-emission scanning electron microscopy (FESEM) images of PrMnO_3_, recorded at different magnifications, reveal an agglomerated assembly of elongated nanocrystallites with rod-like features, forming a porous and rough surface morphology (Fig. 3a). At lower magnification, the material exhibits interconnected secondary aggregates with abundant intergranular voids, while higher-magnification images show anisotropic particles tightly packed within the aggregates. Such morphology is attributed to the citric acid-assisted sol–gel synthesis, where citric acid acts as a chelating agent, forming a homogeneous metal–citrate complex and a polymeric gel network. During thermal decomposition, the combustion of the organic matrix releases gases, generating localised heat and internal pressure that promote homogeneous nucleation, anisotropic growth, and inhibit excessive grain coarsening. The developed porous structure and surface roughness are likely to offer a large number of readily accessible active sites, while also improving light absorption and reactant diffusion, making the material advantageous for photocatalytic applications.^28^

Energy-dispersive X-ray (EDX) spectroscopy was employed to confirm the elemental composition and chemical purity of the prepared PrMnO_3_ sample (Fig. 3b). The spectrum displays only characteristic signals corresponding to Pr (L), Mn (K), and O (K), with atomic percentages of approximately 21.3%, 23.9%, and 54.8%, respectively, in reasonable agreement with the expected stoichiometry considering the limitations of EDX for light elements. The absence of impurity peaks and the uniform elemental distribution across the analysed region indicate successful formation of phase-pure PrMnO_3_. The combination of compositional homogeneity and nanorod-like porous morphology is conducive to efficient charge separation and transport under visible-light irradiation, thereby enhancing the photocatalytic performance of the material.

#### 3.1.5. Surface chemical composition and oxidation state analysis (XPS)

**Fig. 4:**
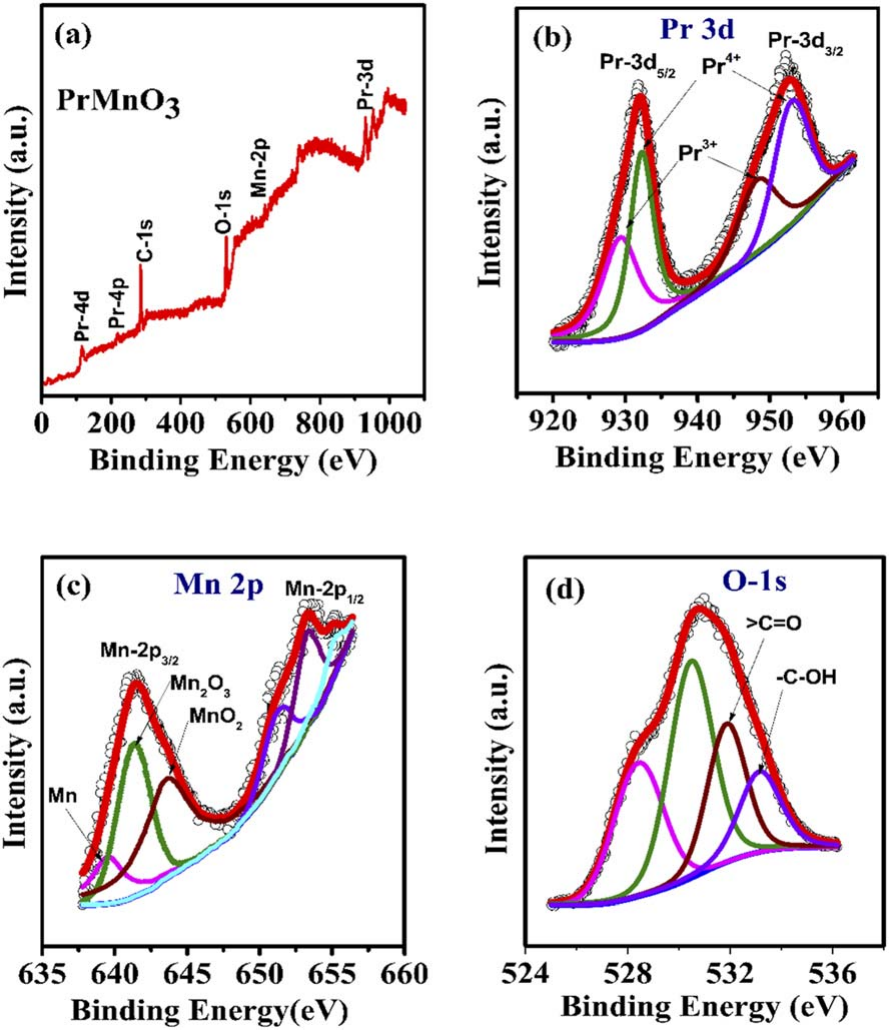
(a). XPS full scan spectra of the PrMnO_3_ material, (b) Pr 3d, (c)Mn 2p, and (d) O 1s.

X-ray photoelectron spectroscopy (XPS) was employed to investigate the surface elemental composition and oxidation states of the PrMnO_3_ perovskite photocatalyst. As illustrated in Fig. 4a, the survey spectrum confirms the successful formation of PrMnO_3_, evidenced by the presence of characteristic Pr 3d, Mn 2p, and O 1s signals, indicating a uniform elemental distribution within the material. The high-resolution Pr 3d spectrum (Fig. 4b) reveals the coexistence of mixed oxidation states of praseodymium, namely Pr^3+^ and Pr^4+^. The deconvoluted peaks corresponding to Pr 3d_5/2_ and Pr 3d_3/2_ are observed at binding energies of approximately 929.4, 932.3, 948.7, and 953.3 eV, corroborating the mixed-valence nature of praseodymium.^29^ The Mn 2p spectrum (Fig. 4c) displays two primary peaks located at 641.3 eV and 653.3 eV, corresponding to Mn 2p_3/2_ and Mn 2p_1/2_, respectively.^30^ The observed spin–orbit splitting of ∼12 eV confirms the presence of manganese in multiple oxidation states.^31^ Further spectral deconvolution reveals peaks at 641.3 and 643.6 eV (Mn 2p_3/2_) and at 653.3 and 655.0 eV (Mn 2p_1/2_), indicating contributions from both Mn^3+^ and Mn^4+^ species. Specifically, the peaks at ∼641.8 and 653.3 eV are attributed to Mn^3+^, whereas those at ∼644.8 and 655.7 eV correspond to Mn^4+^.^32^ The presence of Mn^3+^ is consistent with the expected oxidation state of manganese in PrMnO_3_ nanoparticles.

The O 1s spectrum (Fig. 4d) was deconvoluted into several components corresponding to different oxygen environments, such as lattice oxygen, oxygen vacancies, and surface-adsorbed oxygen. The fitted peaks located at approximately 528.5, 530.5, 531.9, and 533.1 eV were assigned to lattice oxygen (O_L_), defect-related oxygen (oxygen vacancies, O_v_), and chemisorbed oxygen species. Notably, the peaks at ∼528.5 eV and ∼531.9 eV correspond to lattice oxygen and oxygen vacancies, respectively, confirming the presence of defect sites within the PrMnO_3_ framework.^33^ A higher concentration of oxygen vacancies is beneficial for enhancing charge carrier mobility and facilitating rapid ionic diffusion during photocatalytic processes.

Overall, the XPS analysis demonstrates that the PrMnO_3_ material possesses mixed redox-active couples (Pr^3+^/Pr^4+^ and Mn^3+^/Mn^4+^) along with abundant oxygen vacancies. These features synergistically promote efficient charge separation and transfer, thereby significantly enhancing the photocatalytic degradation performance.

#### 3.1.6. Surface area and porosity analysis (BET)

**Fig. 5:**
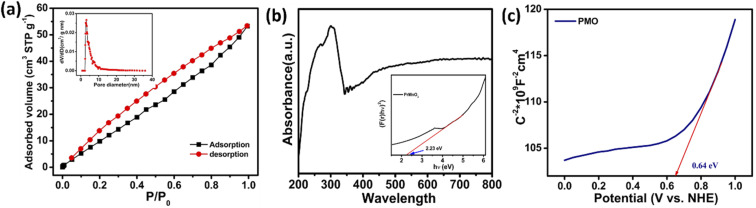
(a) N_2_ adsorption–desorption isotherm of PrMnO_3_ (PMO), with the inset showing the corresponding pore size distribution. (b) UV-vis absorption spectrum of PrMnO_3_; the inset displays the Tauc plot used to determine the optical band gap (∼2.23 eV). (c) Mott–Schottky (M–S) plot of PrMnO_3_.

The N_2_ adsorption–desorption isotherm of the PrMnO_3_ sample (Fig. 5a) displays a characteristic type-IV isotherm with a narrow hysteresis loop, confirming the presence of well-defined mesopores. The steady rise in adsorption volume with increasing relative pressure (*P*/*P*_0_) and the pronounced capillary condensation region further validate the mesoporous architecture. The total adsorbed N_2_ volume approaching ∼50 cm^3^ g^−1^ at *P*/*P*_0_ = 1.0 suggests good surface accessibility and a porous framework.

The BJH pore size distribution (inset) shows a dominant peak below 10 nm, indicating uniform mesopore formation. The sharp decline in intensity with increasing pore diameter reflects a narrow pore size distribution with negligible macropore contribution.

BET analysis revealed that PrMnO_3_ possesses a specific surface area of 70.375 m^2^ g^−1^, a pore volume of 0.0825 cm^3^ g^−1^, and an average pore diameter of 4.694 nm, which falls within the mesoporous range. Such a high surface area combined with small, uniform mesopores enhances molecular diffusion and provides more active sites, making PrMnO_3_ highly suitable for photocatalytic applications (Table 1).

**Table 1:** Textural properties of PrMnO_3_, including specific surface area, average pore diameter, and pore volume

Materials	PrMnO_3_
Surface area (m^2^ g^−1^)	70.375
Average pore diameter (nm)	4.694
Pore volume (cm^3^ g^−1^)	0.0825

#### 3.1.7. Optical properties (UV-vis and band gap)

The optical behaviour of the synthesised PrMnO_3_ nanostructures was examined by UV-visible diffuse reflectance spectroscopy (DRS) (Fig. 5b). To evaluate the optical band gap energy (*E*_g_), the reflectance data were converted into the Kubelka–Munk function, *F*(*R*). The band gap value was then estimated using the Tauc relation:

2 (*F*(*R*)*hν*)^*n*^ = *A*(*hν* − *E*_g_)

where *hν* is the photon energy, *A* is a proportionality constant, and *n* depends on the nature of the electronic transition. In the present study, *n* = 2 was employed, corresponding to a direct allowed transition, as the best linear fitting was obtained for the (*F*(*R*)*hν*)^2^*versus hν* plot.

Extrapolation of the linear region of the Tauc plot to the energy axis yielded a band gap of ∼2.23 eV for PrMnO_3_. Although bulk PrMnO_3_ is often reported as an indirect band gap semiconductor, several studies have demonstrated that nanostructured and porous PrMnO_3_ exhibits direct band gap behaviour, attributed to defect-induced electronic states, lattice distortion, and reduced particle size effects. Comparable direct band-gap behaviour has also been reported for PrMnO_3_-based nanomaterials and composite systems by Anjiraki *et al.*^25^ and Aguilar *et al.*,^28^ where the selection of *n* = 2 was supported by strong visible-light absorption and the good linearity of the corresponding Tauc plots. Hence, the band-gap behaviour observed in the present study is in agreement with earlier reports on PrMnO_3_ nanostructures.

#### 3.1.8. Mott–Schottky analysis

To gain deeper insight into the electronic characteristics of PrMnO_3_, Mott–Schottky analysis was performed to evaluate the flat-band potential (*V*_fb_) and identify the semiconductor type of the material. The Mott–Schottky relationship is given as follows:^34^3
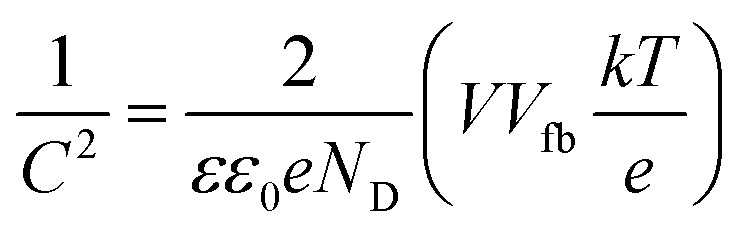
where *C* is the space charge capacitance, *e* denotes the elementary charge, *ε* is the dielectric constant of the semiconductor, *ε*_0_ is the permittivity of free space, *N*_D_ represents the donor density, *V* is the applied potential, *k* is the Boltzmann constant, and *T* is the absolute temperature. By extrapolating the linear region of the Mott–Schottky plot to the potential axis, the flat-band potential of PMO nanoparticles was determined to be 0.64 V *vs.* Ag/AgCl, which corresponds to 0.83 V *vs.* NHE after conversion using the following equation.

4 *E*_NHE_ = *E*_Ag/AgCl_ + 0.197

The flat-band potentials can be approximately considered to be conduction band edges.^35^ In turn, the valence band potentials of PMO are obtained as 3.06 eV, respectively, using the following equation:^36^

5 *E*_CB_ = *E*_VB_ − *E*_g_

A schematic band structure diagram of PMO, along with the relevant redox potentials, is presented in Fig. 5c. As illustrated, the conduction band is positioned at +0.83 V *vs.* NHE, while the valence band lies at +3.06 V *vs.* NHE, clearly depicting the band gap and charge transition under visible-light irradiation. The diagram also highlights the relative positions of key redox potentials, including O_2_/˙O_2_^−^ (−0.33 V *vs.* NHE) and H_2_O/˙OH (+2.40 V *vs.* NHE), enabling direct visualisation of the feasible photocatalytic pathways.

#### 3.1.9. Charge carrier dynamics analysis

**Fig. 6:**
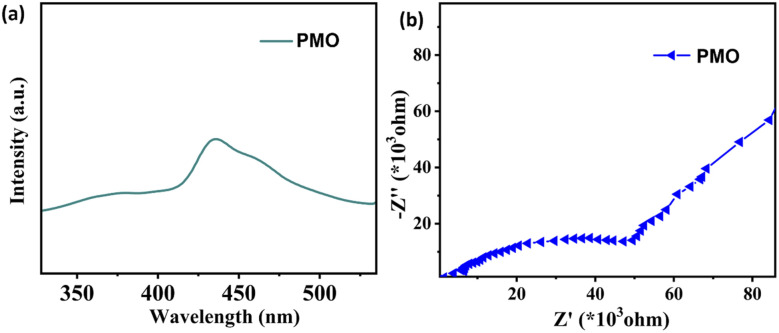
(a) Photoluminescence (PL) spectrum and (b) electrochemical impedance spectroscopy (EIS) Nyquist plot of the synthesised PrMnO_3_ photocatalyst.

To further investigate the charge separation and interfacial charge-transfer behaviour of the synthesised PrMnO_3_ photocatalyst, photoluminescence (PL) spectroscopy and electrochemical impedance spectroscopy (EIS) measurements were carried out, and the results are presented in Fig. 6a and b.

The PL spectrum (Fig. 6a) exhibits a broad emission band centred at approximately 435 nm, originating from the radiative recombination of photogenerated electron–hole pairs. The relatively low emission intensity suggests that radiative recombination is effectively suppressed, allowing a larger fraction of the photogenerated charge carriers to participate in photocatalytic reactions. This behaviour is consistent with the XPS results, which revealed the coexistence of mixed-valence species (Pr^3+^/Pr^4+^ and Mn^3+^/Mn^4+^) together with abundant oxygen vacancies. These defect sites can act as temporary electron-trapping centres, facilitating charge migration and reducing direct electron–hole recombination.

The EIS Nyquist plot (Fig. 6b) displays a relatively small semicircular arc in the high-frequency region followed by a linear tail at lower frequencies. The semicircle corresponds to the interfacial charge-transfer resistance (*R*_ct_), while the linear region is associated with diffusion-controlled charge transport. The relatively low charge-transfer resistance indicates efficient interfacial electron transport and favourable separation of photogenerated charge carriers, thereby promoting visible-light-driven photocatalytic reactions.

The PL and EIS results are in good agreement with the radical trapping experiments and band-structure analysis. The efficient charge separation promoted by mixed-valence species and oxygen vacancies prolongs the lifetime of photogenerated holes, enabling the generation of hydroxyl radicals (˙OH), while photogenerated electrons participate in surface reduction reactions and suppress electron–hole recombination. These findings provide additional experimental evidence supporting the proposed photocatalytic mechanism of PrMnO_3_ under visible-light irradiation.

### 3.2. Photocatalytic degradation performance

#### 3.2.1. Photocatalytic degradation of different antibiotics

**Fig. 7:**
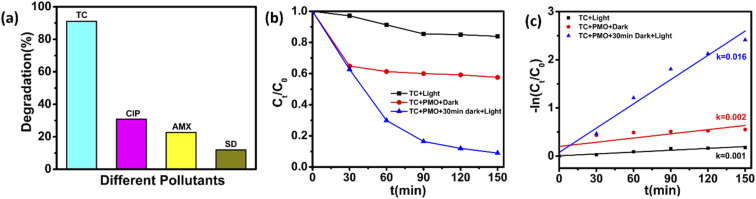
(a) Photocatalytic degradation of TC, CIP, AMX, and SDZ over PrMnO_3_. (b) Degradation profiles of TC under different experimental conditions. (c) Pseudo-first-order kinetic fitting of TC degradation.

To evaluate the photocatalytic performance of the synthesised PMO material toward different antibiotic pollutants, four representative antibiotics, Tetracycline (TC), Ciprofloxacin (CIP), Amoxicillin (AMX) and Sulfadiazine (SDZ), were selected as model organic contaminants. Under visible-light irradiation for 120 min, with an initial pollutant concentration of 20 ppm and a catalyst loading of 50 mg, the observed degradation efficiencies were 91.04% (TC), 30.88% (CIP), 22.56% (AMX), and 11.88% (SDZ). Among these, TC exhibited the highest degradation, demonstrating that PMO has markedly greater photocatalytic activity toward TC than toward CIP, AMX, or SDZ under the same conditions (Fig. 7a).

#### 3.2.2. Control experiments and kinetic analysis

To validate the photocatalytic activity of the synthesised PMO and to assess its efficiency under different conditions, three controlled experiments were performed: (i) TC + visible light, (ii) TC + PMO in the dark, and (iii) TC + PMO under visible light irradiation. All experiments were conducted at a nearly neutral pH (6.62) with an initial TC concentration of 20 ppm and a catalyst dosage of 50 mg. The corresponding degradation behaviour is illustrated in Fig. 7b. In the absence of the catalyst, only ∼12% degradation of TC was observed after 150 min of visible-light irradiation, indicating negligible self-photolysis of the antibiotic. When the catalyst was introduced in the dark, ∼42% removal was observed within the same time interval, indicating that PMO exhibits a considerable adsorption capacity toward TC molecules. Notably, when the system was equilibrated in the dark for 30 min followed by 120 min of visible-light irradiation, the degradation efficiency markedly increased to 91.04%, confirming the synergistic contribution of initial adsorption and subsequent photocatalytic oxidation in achieving enhanced removal performance. The reaction kinetics were further examined using the pseudo-first-order model expressed as:^37^6
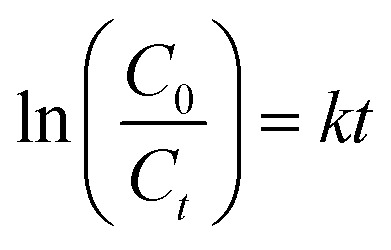
where *C*_0_ is the initial TC concentration, *C*_*t*_ is the concentration at time *t*, and *k* is the apparent rate constant. As shown in Fig. 7c, the kinetic plots displayed a strong linear correlation, confirming that TC degradation over PMO follows pseudo-first-order kinetics. The calculated rate constant under PMO + visible light conditions was 0.016 min^−1^, which is markedly higher than those obtained under light alone (0.001 min^−1^) and dark adsorption (0.002 min^−1^). This enhanced rate implies that pre-adsorption of TC on the catalyst surface facilitates more efficient charge transfer and reactive species generation upon illumination.

Overall, these results demonstrate that PMO is an efficient visible light-responsive photocatalyst, capable of rapidly degrading persistent antibiotic pollutants such as tetracycline, and holds strong potential for wastewater treatment applications.

#### 3.2.3. Effect of catalyst dosage

**Fig. 8:**
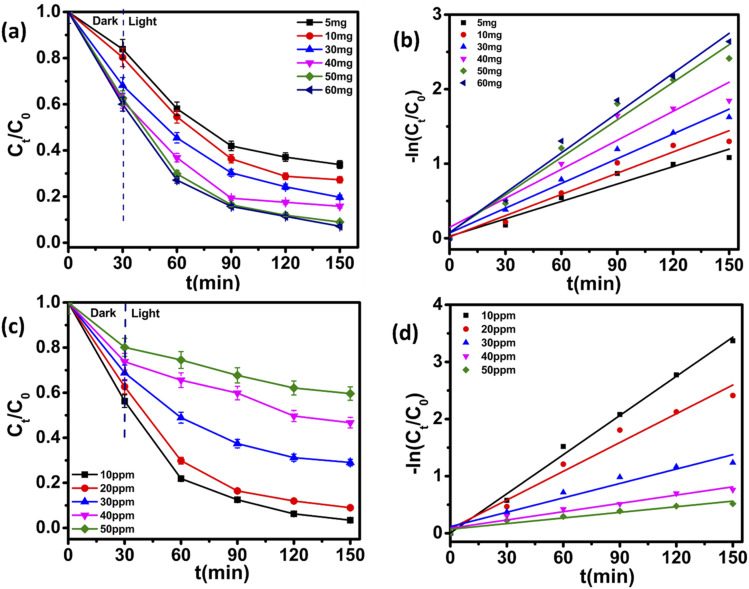
(a) Effect of PMO dosage on TC degradation. (b) Corresponding pseudo-first-order kinetic plots. (c) Effect of initial TC concentration on TC degradation. (d) Corresponding pseudo-first-order kinetic plots.

The influence of PMO photocatalyst dosage on TC degradation was systematically evaluated by varying the catalyst amount from 5 to 60 mg, while maintaining a constant initial TC concentration of 20 ppm in 100 mL solution (Fig. 8a and b). As anticipated, the degradation efficiency increased with increasing catalyst dosage, which can be attributed to the greater availability of surface-active sites and the enhanced generation of reactive oxygen species, thereby facilitating more efficient interaction between the photocatalyst and TC molecules.^38^ Notably, the degradation efficiency and rate constant improved from 66.12% (*k* = 0.007 min^−1^) to 91.04% (*k* = 0.016 min^−1^) when the catalyst dosage increased from 5 to 50 mg, confirming the crucial role of the photocatalyst in accelerating the decomposition process. However, a further increase in catalyst dosage beyond 50 mg did not yield a significant improvement in photocatalytic efficiency. This phenomenon may be attributed to excess catalyst particles, which cause light scattering and shielding, reducing the penetration of visible light into the reaction suspension.^39^ Consequently, the formation of photoinduced electron hole pairs is restricted, diminishing the photocatalytic performance.^40^ Therefore, 50 mg of PMO was identified as the optimum dosage and subsequently employed for further photocatalytic studies.

#### 3.2.4. Effect of initial TC concentration

The effect of initial tetracycline concentration on its photocatalytic degradation over PMO is presented in Fig. 8c and d and in Table 2. A clear decrease in photocatalytic efficiency was observed with increasing pollutant concentration. At lower concentrations (10 and 20 ppm), the degradation was highly efficient, with *C*/*C*_0_ values decreasing to approximately 0.03 and 0.08, respectively, after 120 min of visible-light irradiation. In contrast, further increases in concentration (30–50 ppm) resulted in progressively weaker degradation performance, with *C*/*C*_0_ values remaining around 0.30, 0.40, and above 0.60 for 30, 40, and 50 ppm, respectively. This decline can be attributed to the progressive saturation of the active surface sites of PMO at elevated TC concentrations. Excess TC molecules competitively occupy adsorption and photocatalytic sites, thereby restricting the generation and diffusion of photogenerated charge carriers and limiting the availability of reactive oxygen species (ROS) required for efficient degradation.^41^ As a result, the photocatalytic reaction becomes mass-transfer-limited, leading to lower degradation efficiencies at higher pollutant loads.

These results indicate that lower initial TC concentrations promote more efficient catalyst–pollutant interactions and faster degradation kinetics. In contrast, higher concentrations suppress photocatalytic activity due to site saturation and reduced ROS accessibility.

**Table 2:** Relationship between the initial TC concentration and the pseudo-first-order rate constant

SI. no.	TC concentration (ppm)	*R* ^2^	Rate constant, *k* (min^−1^)
1	10	0.99	0.022
2	20	0.96	0.016
3	30	0.94	0.008
4	40	0.94	0.004
5	50	0.92	0.003

#### 3.2.5. Effect of pH on TC degradation

**Fig. 9:**
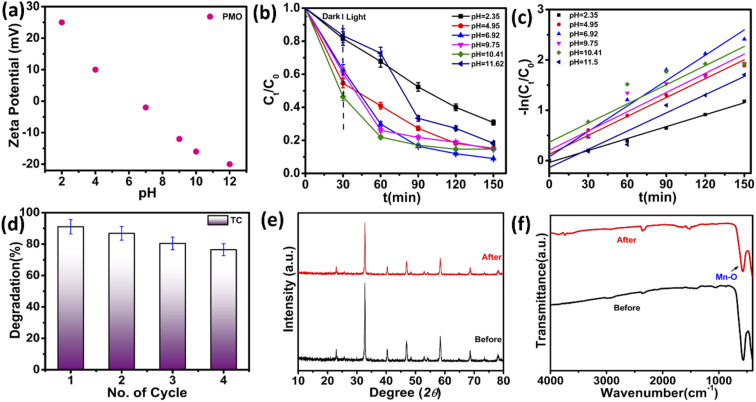
(a) Zeta potential of PrMnO_3_. (b) Effect of initial pH on the photocatalytic degradation of TC. (c) Corresponding pseudo-first-order kinetic plots. Reaction conditions: PMO = 50 mg, TC = 20 mg L^−1^, visible-light irradiation for 120 min. (d) Recycling performance of PrMnO_3_ for TC degradation. (e) XRD patterns and (f) FTIR spectra of PrMnO_3_ before and after repeated photocatalytic cycles.

The surface charge behaviour of PMO was examined through zeta potential measurements at different pH values (2–12), as presented in Fig. 9a. The zeta potential gradually shifts from positive to negative as pH increases. It approaches a near-neutral value around pH ∼6–7, indicating that the point of zero charge (pH_pzc_) lies in this region. This variation in surface charge plays a key role in governing the interaction between the catalyst surface and tetracycline molecules.

The photocatalytic performance of PMO was further evaluated over a wide pH range (2.35–11.62), and the corresponding degradation profiles are shown in Fig. 9b, while the kinetic plots (ln(*C*_0_/*C*_*t*_) *vs.* time) are presented in Fig. 9c. The calculated pseudo-first-order rate constants are summarised in Table 3. It is well understood that pH influences photocatalytic reactions by affecting (i) the surface charge of the catalyst, (ii) the speciation of TC, and (iii) the generation of reactive oxygen species such as ˙OH radicals.

**Table 3:** Influence of solution pH on TC degradation efficiency and kinetic rate constant

pH	% Degradation	*C*_*t*_/*C*_0_	*K* (min^−1^)
2.35	69.23	0.30	0.007
4.95	84.84	0.15	0.012
6.92	91.04	0.08	0.016
9.75	85.50	0.14	0.012
10.41	85.36	0.14	0.012
11.62	81.81	0.18	0.012

At strongly acidic conditions (pH 2.35), the degradation efficiency was relatively low (69.23%, *k* = 0.007 min^−1^). This reduced activity can be linked to the positively charged surface of PMO under acidic conditions, which likely repels protonated TC species. In addition, the high concentration of H^+^ ions may suppress the formation of surface hydroxyl groups, thereby limiting the generation of reactive species.

As the pH increases, a clear improvement in degradation efficiency is observed. At pH 4.95, the degradation reaches 84.84% (*k* = 0.012 min^−1^), and further increases to a maximum of 91.04% (*k* = 0.016 min^−1^) at pH 6.92. This enhanced performance near neutral pH can be attributed to the near-neutral surface charge of PMO (close to pH_pzc_), which reduces electrostatic barriers and facilitates better interaction with TC molecules. At the same time, TC predominantly exists in its zwitterionic form in this pH range, which is favourable for adsorption and subsequent photocatalytic degradation.

At alkaline pH (9.75–10.41), the degradation efficiency shows a slight decrease but remains relatively high (∼85%, *k* ≈ 0.012 min^−1^). The presence of OH^−^ ions in this region can promote the formation of ˙OH radicals, which supports the degradation process. However, the negatively charged catalyst surface at higher pH may lead to some degree of electrostatic repulsion with deprotonated TC species, resulting in a marginal reduction in efficiency.

At strongly alkaline conditions (pH 11.62), the degradation efficiency further decreases to 81.81%, which may be due to reduced adsorption of TC and increased competition from excess OH^−^ ions for active sites on the catalyst surface.

Overall, PMO exhibits stable and efficient photocatalytic performance over a broad pH range (4.95–10.41), with optimal activity observed near neutral pH. This behaviour highlights its suitability for practical wastewater treatment, where pH conditions may vary.^42–44^

#### 3.2.6. Stability, reusability, and structural integrity of PrMnO_3_ photocatalyst

The long-term stability and reusability of the PrMnO_3_ perovskite photocatalyst were systematically evaluated through consecutive photocatalytic degradation cycles of TC, as illustrated in Fig. 9a. After each photocatalytic run, the catalyst was recovered by centrifugation, thoroughly washed with deionised water to remove adsorbed intermediates, and reused under the same reaction conditions. This procedure was adopted to simulate practical operating conditions and to evaluate the robustness of PMO during prolonged photocatalytic operation. As illustrated in Fig. 9d, PMO exhibited remarkable cycling stability, maintaining a high TC degradation efficiency even after three successive photocatalytic cycles. Only a minor decrease (∼6%) in degradation efficiency was observed after the third cycle, which was primarily due to unavoidable catalyst loss during recovery and to partial surface coverage by strongly adsorbed degradation intermediates.^45^ Importantly, the absence of any abrupt decline in photocatalytic activity suggests that PMO retains its photocatalytic performance during repeated use.

To further elucidate the structural and chemical stability of PMO, XRD and FTIR analyses were performed on the fresh and recycled catalysts (Fig. 9e and f). The XRD patterns of the reused PMO showed no noticeable shift in diffraction peak positions, nor the emergence of secondary phases, confirming that the perovskite crystal structure and phase purity were retained after repeated photocatalytic reactions. These results indicate that no significant phase transformation or structural degradation of the PrMnO_3_ catalyst occurred during the photocatalytic reaction. The FTIR spectra recorded before and after TC degradation provide additional insight into the surface chemical stability of PMO (Fig. 9f). In both spectra, the characteristic metal–oxygen vibration bands associated with the Mn–O lattice framework appear in the low-wavenumber region (typically below 700 cm^−1^), confirming the preservation of the perovskite backbone after photocatalysis. No obvious new absorption bands corresponding to organic functional groups were observed in the recycled catalyst. This suggests that no substantial accumulation of degradation products occurred on the catalyst surface after photocatalysis. However, FTIR analysis alone cannot completely exclude the presence of trace adsorbed intermediates because of its limited sensitivity. The slight reduction in transmittance intensity observed in the FTIR spectrum of the recycled PMO may be attributed to weakly adsorbed intermediates or surface hydroxyl modifications generated during the photocatalytic process. However, the absence of peak shifts or new vibrational modes suggests that these surface changes are reversible and do not compromise the fundamental chemical structure of PMO.^46,47^ Collectively, the XRD, FTIR, and recycling studies indicate that PrMnO_3_ retains its crystal structure and photocatalytic activity after repeated use, demonstrating its good structural stability and reusability for photocatalytic degradation.

#### 3.2.7. Photocatalytic performance in real water matrices

**Fig. 10:**
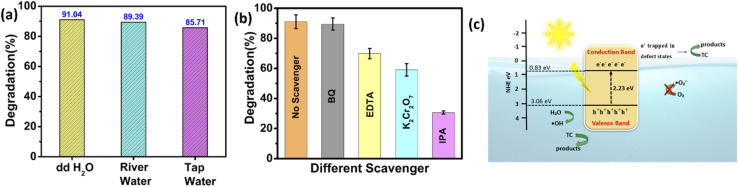
(a) Photocatalytic degradation of TC over PrMnO_3_ in different water matrices (deionized water, Mahanadi River water, and Ravenshaw University tap water). (b) Effect of different scavengers on the photocatalytic degradation of TC over PrMnO_3_. Reaction conditions: TC = 20 mg L^−1^, PMO = 50 mg, visible-light irradiation for 120 min. Scavengers: IPA (˙OH), EDTA (h^+^), K_2_Cr_2_O_7_ (e^−^), and BQ (˙O_2_^−^). (c) Schematic illustration of the proposed charge-transfer mechanism over PrMnO_3_ under visible-light irradiation.

To assess the practical applicability of the synthesised photocatalyst, visible-light-driven degradation experiments were carried out in different water media, namely double-distilled water (dd H_2_O), tap water collected from Ravenshaw University, and surface water obtained from the Mahanadi River (Fig. 10a). For each experiment, a tetracycline solution (20 ppm) was prepared in the respective water matrix, and 50 mg of PrMnO_3_ photocatalyst was used under visible-light irradiation for 120 min.

As presented in Fig. 10a, the highest degradation efficiency of 91.04% was achieved in dd H_2_O, whereas slightly lower efficiencies of 89.39% and 85.71% were recorded in river water and tap water, respectively. The modest decline in photocatalytic activity in real-water samples is likely due to the presence of dissolved inorganic ions and natural organic matter, which can compete for surface-active sites or scavenge reactive species generated during the photocatalytic process.

Despite this slight reduction, the PrMnO_3_ photocatalyst maintains high degradation efficiency (>85%) across all tested water matrices, demonstrating its robustness and strong resistance to matrix interference. These results confirm the effectiveness of the visible-light-driven PrMnO_3_ system under realistic environmental conditions and highlight its potential for practical applications in wastewater treatment, particularly for removing antibiotic contaminants from complex aqueous systems.

### 3.3. Photocatalytic mechanism

#### 3.3.1. Active species trapping experiments

Radical-scavenging experiments were carried out to identify the dominant reactive species involved in the visible-light-assisted degradation of tetracycline, as shown in Fig. 10b. In the absence of a scavenger, PMO achieved a high degradation efficiency of 91.04%, demonstrating its strong photocatalytic performance.

When isopropyl alcohol (IPA), a commonly used scavenger of hydroxyl radicals (˙OH), was introduced into the reaction system, the degradation efficiency dropped significantly to 30.63%, indicating that ˙OH radicals are the major contributors to the degradation process. Likewise, the addition of ethylenediaminetetraacetic acid (EDTA), which acts as a hole (h^+^) scavenger, lowered the degradation efficiency to 69.84%, suggesting that photogenerated holes also play an active role in the oxidation pathway.

When K_2_Cr_2_O_7_, an electron (e^−^) scavenger, was introduced, the degradation efficiency decreased to 58.96%. This substantial inhibition indicates that photogenerated electrons play an essential role in maintaining efficient charge separation. By scavenging conduction-band electrons, K_2_Cr_2_O_7_ reduces the availability of charge carriers participating in interfacial reduction reactions and increases the probability of electron–hole recombination. Consequently, the lifetime of photogenerated holes available for the generation of hydroxyl radicals (˙OH) is shortened, leading to a significant decrease in photocatalytic degradation efficiency.

In contrast, the addition of *p*-benzoquinone (BQ), a superoxide radical (˙O_2_^−^) scavenger, resulted in only a slight reduction in degradation efficiency (89.53%), suggesting that ˙O_2_^−^ radicals contribute minimally to TC degradation. This observation agrees well with the band structure of PMO, where the conduction band potential is insufficiently negative to efficiently reduce dissolved oxygen into ˙O_2_^−^ radicals.^38–49^

Overall, the scavenger experiments demonstrate that the photocatalytic degradation of TC over PrMnO_3_ is predominantly governed by ˙OH radicals, with photogenerated holes serving as an additional active oxidising species, while photogenerated electrons play an indirect yet essential role by maintaining charge separation and suppressing electron–hole recombination. The contribution of ˙O_2_^−^ radicals is negligible.

#### 3.3.2. Proposed charge transfer mechanism

The above scavenging results are in strong agreement with the electronic band structure determined from Mott–Schottky (M–S) and UV-vis analyses. The band structure of PrMnO_3_ was constructed by combining UV-vis-derived band gap values with Mott–Schottky-derived conduction band positions (Fig. 10c), providing insight into the electron transfer mechanism during photocatalysis. As discussed in Section 3.1.8, the flat-band potential of PMO was estimated to be 0.83 V *vs.* NHE, which is close to the conduction band edge (E_CB_) for an n-type semiconductor.^50^ Based on the optical band gap of 2.23 eV, the valence band edge (E_VB_) was calculated to be 3.06 V *vs.* NHE.

Upon visible light irradiation, PMO generates electron–hole pairs as follows:

7 PrMnO_3_ + *hν* → PrMnO_3_(e_CB_− + h_VB_+)

The photocatalytic mechanism is well supported by the band structure determined from the UV-vis and Mott–Schottky analyses. Upon visible-light irradiation, electrons are excited from the valence band (VB) to the conduction band (CB), generating electron–hole pairs.

Because the valence band potential (3.06 V *vs.* NHE) is considerably more positive than the oxidation potential of H_2_O/˙OH (2.40 V *vs.* NHE), photogenerated holes possess sufficient oxidation power to convert adsorbed water molecules or surface hydroxyl groups into highly reactive hydroxyl radicals.

8 h_VB_^+^ + H_2_O → ˙OH + H^+^

9 h_VB_^+^ + OH^−^ → ˙OH

10 ˙OH/h^+^ + TC → degradation intermediates → low molecular weight products

The generated ˙OH radicals, together with photogenerated holes, subsequently oxidise tetracycline into smaller intermediates through successive oxidation and ring-opening reactions.

In contrast, the conduction band potential (0.83 V *vs.* NHE) is more positive than the O_2_/˙O_2_^−^ reduction potential (−0.33 V *vs.* NHE), making the formation of superoxide radicals thermodynamically unfavourable. This is in excellent agreement with the scavenger experiments, where BQ produced only a negligible decrease in photocatalytic efficiency.

Although conduction-band electrons do not efficiently generate ˙O_2_^−^ radicals, they remain crucial for maintaining effective charge separation. The presence of conduction-band electrons reduces the probability of direct electron–hole recombination, thereby prolonging the lifetime of photogenerated holes. These long-lived holes continuously generate abundant ˙OH radicals, resulting in enhanced photocatalytic degradation.

Therefore, TC degradation over PrMnO_3_ proceeds mainly through a ˙OH radical- and hole-mediated oxidation pathway, while photogenerated electrons indirectly promote photocatalysis by suppressing charge recombination rather than acting as the principal reactive species.

#### 3.3.3. Intermediates and plausible degradation pathways of tetracycline

**Fig. 11:**
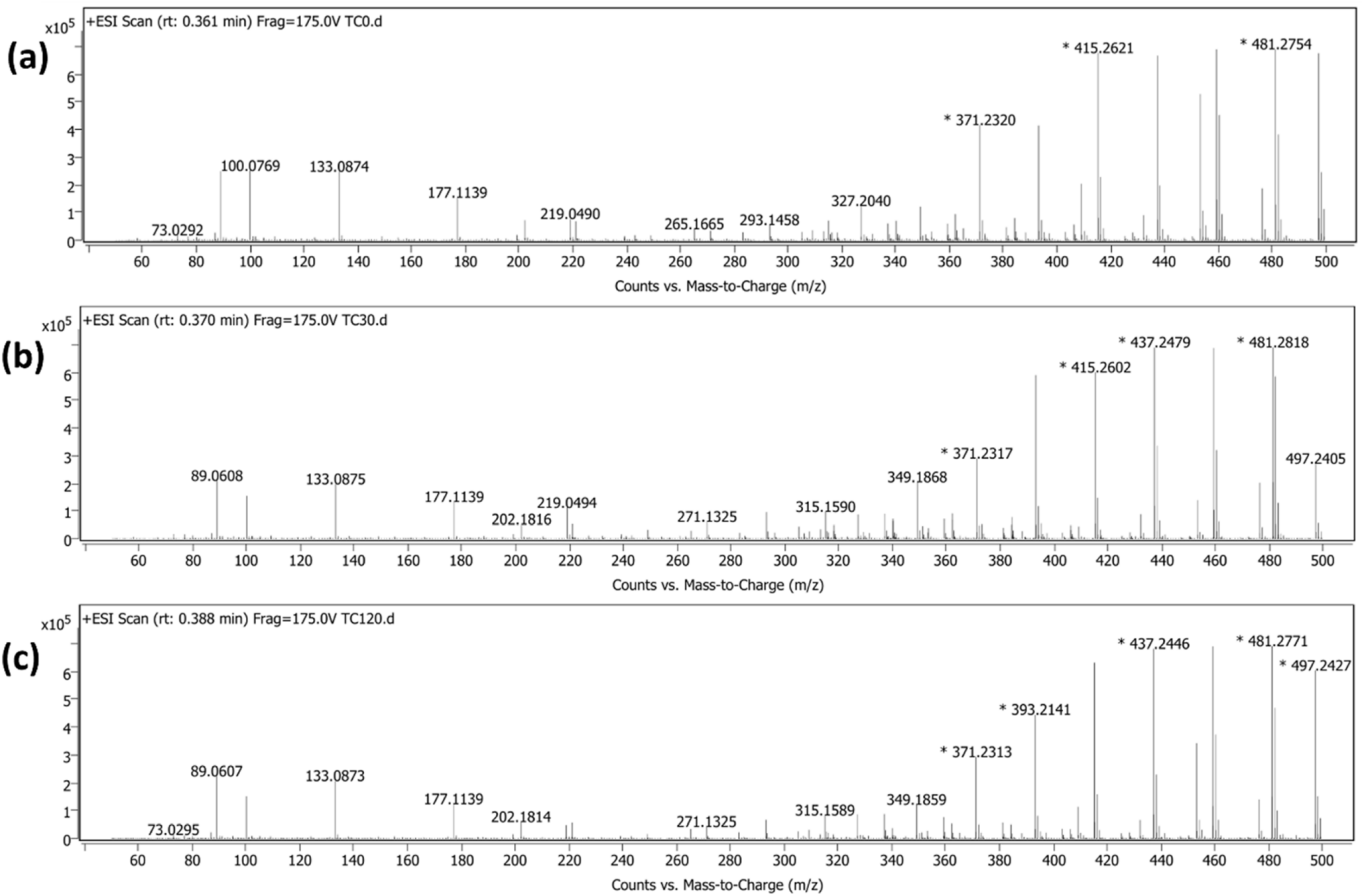
LC-MS spectra of tetracycline (TC): (a) TC at 0 min (without catalyst), (b) TC after 30 min of visible-light irradiation in the presence of PMO, and (c) TC after 120 min of visible-light irradiation in the presence of PMO.

**Fig. 12:**
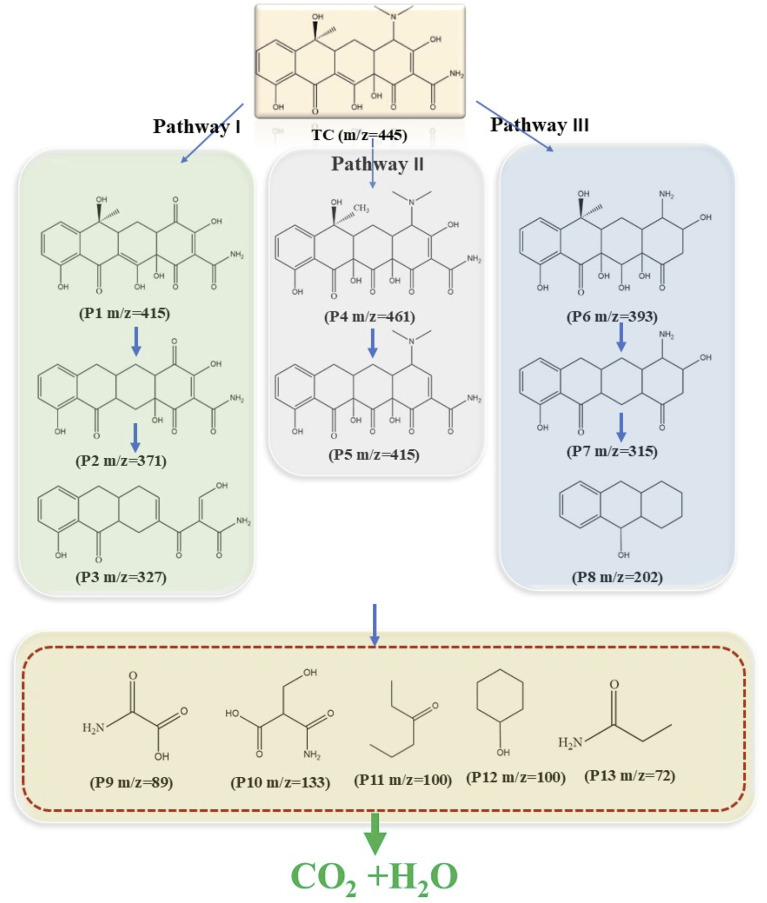
Possible photocatalytic degradation pathways of TC in the presence of PrMnO_3_ catalyst.

To gain deeper insight into the photocatalytic degradation mechanism of tetracycline (TC), liquid chromatography-mass spectrometry (LC-MS) analysis was carried out to identify the intermediate products formed during degradation over the PrMnO_3_ catalyst. The LC-MS spectra of the detected intermediates are presented in Fig. 11. Based on the LC-MS results obtained in this work and previously reported studies; three plausible degradation pathways (Fig. 12) are proposed for the photocatalytic transformation of TC in the presence of the PrMnO_3_ catalyst.

In Pathway I, the *N*,*N*-dimethyl substituent of TC undergoes oxidative transformation, leading to the formation of a species tentatively assigned as intermediate P1 (*m*/*z* = 415). Similar intermediates have been reported in previous studies.^51,52^ As the photocatalytic reaction progressed, P1 underwent successive dealkylation and oxidative fragmentation, yielding the intermediate P2 (*m*/*z* = 371).^53^ Subsequent transformation of P2 through ring-opening and oxidative cleavage reactions resulted in the formation of P3 (*m*/*z* = 327).^54^

In Pathway II, TC was attacked by reactive oxygen species (ROS), resulting in hydroxylation and oxidative transformation to generate intermediate P4 (*m*/*z* = 461). Comparable species have previously been identified as primary intermediates during TC degradation.^55,56^ Further dehydroxylation and dealkylation of P4 led to the formation of P5 (*m*/*z* = 371).^57^

In Pathway III, TC (*m*/*z* = 445) was initially attacked by reactive radical species, leading to sequential demethylation and deamidation, and the formation of intermediate P6 (*m*/*z* = 393).^53^ Further transformation of P6 through successive dehydroxylation and demethylation steps generated P7 (*m*/*z* = 315). Subsequently, P7 underwent additional deamination, ring-cleavage, and oxidative fragmentation processes, ultimately yielding the lower-molecular-weight intermediate P8 (*m*/*z* = 202).^58^

As the reaction proceeded, these intermediate species underwent further oxidation and ring-cleavage reactions, producing lower-molecular-weight oxidation products through successive degradation steps.

#### 3.3.4. Comparison with previously reported photocatalysts

To place the photocatalytic performance of the synthesised PrMnO_3_ in context, its activity was compared with representative visible-light-responsive photocatalysts previously reported for tetracycline degradation (Table 4). The comparison includes catalyst dosage, initial tetracycline concentration, irradiation time, and degradation efficiency, enabling a more comprehensive evaluation under different experimental conditions. Although direct comparison among photocatalytic systems should be interpreted cautiously because of differences in catalyst composition, reactor configuration, light intensity, and operating conditions, the present PrMnO_3_ photocatalyst exhibited competitive performance, achieving 91.04% degradation of 20 mg per L tetracycline within 120 min using a catalyst dosage of 0.5 g L^−1^. Notably, unlike many previously reported photocatalysts that rely on heterojunctions, composite structures, or noble-metal modifications to achieve enhanced visible-light activity, the present study demonstrates that pristine phase-pure PrMnO_3_ can efficiently degrade tetracycline under visible-light irradiation. Furthermore, the combination of comprehensive physicochemical characterisation with active-species trapping and LC-MS analyses provides valuable insight into the structure–property–performance relationship and the photocatalytic degradation mechanism. These findings highlight the potential of pristine PrMnO_3_ as a simple yet effective visible-light photocatalyst for the removal of antibiotic contaminants from water.

**Table 4:** Comparison of PrMnO_3_ with reported photocatalysts for tetracycline degradation

SI no.	Catalyst	Catalyst (g L^−1^)	Tetracycline (mg L^−1^)	Duration (min)	Degradation (%)	Ref.
1	CeMnO_3_	0.3	10	90	89	59
2	BiFeO_3_/TiO_2_	1.0	10	180	72	60
3	Bi/BiVO_4_	0.5	10	60	74.7	61
4	LaCoO_3_/Bi_4_Ti_3_O_12_	0.25	5	100	87.8	62
5	LFO/PU	—	10	180	94	63
	CFO/PU	—	10	180	80	
6	8 wt% NiFe-LDH/BiOBr	1.0	10	120	83	64
7	V_2_O_5_/NH_2_-MIL-101(Fe)	0.5	—	120	88.3	65
8	Porous g-C_3_N_4_	1.0	10	120	83	66
9	GdFeO_3_/NiO@g-C_3_N_4_	0.2	10	45	92.42	67
10	MoO_3_/g-C_3_N_4_	0.3	20	90	81	68
11	PrMnO_3_	0.5	20	120	91.04	This work

## 4. Conclusion

In this study, phase-pure PrMnO_3_ was successfully synthesised *via* a sol–gel method and demonstrated as an efficient visible-light-responsive photocatalyst for tetracycline degradation. The synthesised PrMnO_3_ exhibited a relatively high specific surface area (70.375 m^2^ g^−1^), a narrow band gap (2.23 eV), and mixed-valence states (Pr^3+^/Pr^4+^ and Mn^3+^/Mn^4+^) with oxygen vacancies, which collectively promoted visible-light absorption, efficient charge separation, and enhanced photocatalytic activity. Under the optimised conditions, PrMnO_3_ achieved 91.04% tetracycline degradation within 120 min and exhibited good stability and reusability with only a slight decrease in activity after repeated cycles. Mechanistic investigations, including active-species trapping experiments, Mott–Schottky band structure analysis, and LC-MS identification of degradation intermediates, revealed that hydroxyl radicals (˙OH) and photogenerated holes (h^+^) are the dominant reactive species responsible for tetracycline degradation. This work represents one of the few systematic investigations of pristine PrMnO_3_ for visible-light-driven antibiotic degradation and provides important insights into the structure–property–performance relationship of underexplored Pr-based perovskite photocatalysts. The findings demonstrate the potential of PrMnO_3_ as a promising and sustainable photocatalyst for antibiotic-contaminated wastewater treatment.

## Author contributions

Lipika Nayak: conceptualisation, methodology, investigation. Tusharkanta Hati: methodology, investigation, formal analysis. Akash Panda: methodology, formal analysis. Soumya Ranjan Patra: formal analysis (characterisation). Nigamananda Das: writing – original draft, writing – review and editing, validation. Purnendu Parhi: supervision, visualisation, validation, writing – review and editing.

## Conflicts of interest

The authors declare that they have no conflicts of interest.

## Data Availability

All experimental details, synthesis procedures, and characterization data required for reproducibility have been provided in the main manuscript file.
